# Induction in the Study of Disease
*Lord Bacon's Novum Organum, and other works. Montagu's edition. London: 1834.


**Published:** 1857-01

**Authors:** 


### JOURNAL OF PUBLIC HEALTH.

JANUARY 1857.

### INDUCTION IN THE STUDY OF DISEASE.*

THE method of learning facts and principles by the process of induction, although considered as originating with the labours of Lord Bacon, is really of much more ancient date, and is, in fact, coeval with the origin of philosophy. There is an inductive system, as Mr. Hallam points out, in the writings of Aristotle and the ancients; a fact which Bacon himself recognised, and from which he tried carefully to separate his own design.

The ancient induction was based on the principle of "an inference from a perfect enumeration of particulars to a general law of the whole"; a very comprehensive and accurate view, and, if impracticable, mainly so from its very comprehensiveness.

Bacon, on the contrary, in his system of induction, laid down the principle of deducing universal principles from particular, experimental, or even accidental observations. He based his authority for this step on the assumption that in nature everything is uniform and stable; that whatever has once occurred in nature will, under the same conditions and circumstances, inevitably occur again, and present the same phenomena. This, in a nutshell, is the essence of the Baconian system of inductive philosophy; and thus it is defined, in one brief sentence, in the hundred and fifth aphorism of the first book of the *Novum Organum.*

"The induction", so runs the aphorism, "which proceeds from simple enumeration is puerile, leads to uncertain conclusions, and is exposed to danger from one contradictory instance, deciding generally from too small a number of facts, and those only the most obvious. But a really useful induction for the discovery and demonstration of the sciences, should separate nature by proper rejections and exclusions,

* Lord BACON's Novum Organum, and other works. Montagu's edition. London: 1834.

and then conclude for the affirmative after collecting a sufficient number of negatives."

Again he observes, in aphorism 19, book i: "There are and can exist but two modes of investigating and discovering truth. The one hurries on rapidly from the senses and particulars to the most general axioms, and from them as principles, and their supposed indisputable truth, derives and discovers the intermediate axioms. This is the way now in use. The other constructs its axioms from the senses and particulars, by ascending continually and gradually, till it finally arrives at the most general axioms, which is the true but unattempted way."

In another place, aphorism 24, book i, he refers to the folly of axioms determined upon in argument, "since these can never assist in the discovery of new effects; for the subtility of nature is vastly superior to that of argument. But axioms properly and regularly abstracted from particulars, easily point out and define new particulars, and therefore impart activity to the sciences."

Thus this distinguished philosopher invented, as he presumed, a new mode of induction; and his other writings, relating to science, are intended mainly to fill up the details of the plan he had propounded. But it would be false either to presume that Bacon was right in entirely throwing overboard the old system, or that he was strictly original in inventing the new. He laid down on paper a law which is inherent in mind, and out of this law he constructed a plan of research, of all plans the best adapted for the useful contemplation of nature.

But many men previous to the time of Bacon, and many since who have never read his works, were and are intuitively inductive reasoners after the Baconian plan. Whoever will read carefully the writings of Hippocrates or Plato, will find ample evidence of this line of research. Harvey, the cotemporary, but by no means the admirer, of the lawyer philosopher, and who said sneeringly "that Bacon wrote philosophy like a lord chancellor", was one of the clearest inductive reasoners who have ever appeared; while Kepler, also a cotemporary, but who worked purely by the guidance of his own genius, wrought out the ellipse of the planetary bodies by a grand application of the exclusion process. It was the same with Galileo, who, when investigating with his new telescope the planetary bodies at a period prior to the appearance of Lord Bacon's works, viz., about 1609, worked out solely by induction the fact that the bodies near to Jupiter were satellites of that planet, and not fixed stars of a small magnitude. For, observing them night after night, he saw them move, and take positions which were incompatible with the idea of fixed stars; and thus progressing from one particular fact to another, excluding impossibles, and collecting negatives, he gained the affirmative that the bodies he saw were the satellites of the planet.

Paracelsus, also, was constantly deducing inferences by casting out impossible causes of natural phenomena; and, in our own day, we are constantly meeting with shrewd men amongst the uneducated classes, who think or reason out conclusions in the most natural manner, by removing out of the field of observation such influences as have, on inquiry, no bearing on the phenomenon under consideration.

These facts, however, in no way detract from the honour that is due to Lord Bacon. No man can invent any principle or law in nature; but some men may discover a law, mark it out, and lay it so plainly before the world, that the dullest may recognise it: and this is what Lord Bacon did for his race, in reference to the principle of induction in philosophy—nothing more.

To see clearly how important was the mission of this philosopher, we have but to observe how indifferently those sciences have progressed toward certainty in regard to the discovery of causes and effects, which have as yet not recognised the meaning and end of the instructions which he proffered. In our own medical science, this fact stands out strikingly. We have no steady, onward, sweeping progress, tending to one point in medicine; and we cannot have it, until we remodel our proceedings, accept the inductive system, and acknowledge a natural philosophy.

This fact is true of medicine as a whole, but specially of that part of it which relates to the study of epidemics. We do not say that here we make no discoveries, for that is scarcely the fact; but such discoveries as are made are stumbled upon, rather than wrought out. A serious admission this is, perchance, and one not palatable to our pride or our wanton wandering industry, but none the less truthful on that account.

Bearing upon the subject of the manner in which men too often investigate the great phenomena of every day life, Humboldt has some deep thoughts which apply most appositely to the pursuit of medical discovery.

"The history of science teaches us the difficulties which have opposed the progress of an active spirit of inquiry.

Inaccurate and imperfect observations have led, by false inductions, to the great number of physical views that have been presented as popular prejudices among all classes of society. Thus, by the side of a solid and scientific knowledge of natural phenomena, there has been preserved a system of the pretended results of observation, which is so much the more difficult to shake, as it denies the validity of the facts by which it may be refuted. This empiricism, the melancholy heritage transmitted to us from former times, invariably contends for the truth of its axioms with the arrogance of a narrow-minded spirit. Physical philosophy, on the other hand, when based upon science, doubts because it seeks to investigate, distinguishes between that which is certain and that which is merely probable, and strives incessantly to perfect theory by extending the circle of observation.

"This assemblage of imperfect dogmas bequeathed by one age to another,—this physical philosophy which is composed of popular prejudices,—is not only injurious because it perpetuates error with the obstinacy engendered by the observations of ill observed facts, but also because it hinders the mind from attaining to higher views of nature. Instead of seeking to discover the mean or medium point, around which oscillate, in apparent independence of forces, all the phenomena of the eternal world, this system delights in multiplying exceptions to the law, and seeks, amid phenomena and in organic forms, for something beyond the marvel of a regular succession, and an internal and progressive developement. Ever inclined to believe that the order of nature is disturbed, it refuses to recognise in the present any analogy with the past, and, guided by its own varying hypothesis, seeks at hazard for the cause of these pretended perturbations."

This false line of inquiry, thus forcibly delineated by Humboldt as opposed to the progress of the physical sciences as a whole, is specially obstructive to the science of medicine.

In medicine we have never as yet accepted these simple truths upon which the Baconian philosophy is founded, viz., that in nature everything is uniform; that that which has once occurred will, under the same circumstances and conditions, but under no other circumstances and conditions, occur again. On the contrary, to take an example, we start at the approach of a spreading disease as though the order of nature were disturbed; we look upon the phenomena involved as the ignorant look on an eclipse or on a thunderstorm; we recognise in the present no strict analogy with the past, and, guided by our own varying hypotheses, we seek at hazard for the causes.

We take it, therefore, that the first application of the Inductive Philosophy to the study of any disease, consists in recognising in a disease an uniformity and a consistency of nature.

Nor do the facts that in the visitations of disease humanity suffers, and that in the physical and sensation-built body of man the phenomena of the natural event are developed, in any way modify this position; but they rather strengthen it, because they bring the study implied in the inquiry nearer home and conjoin it to dearer interests. For, as we are bound to accept a disease as a natural fact, as fully as we are necessitated to accept an eclipse as a fact of the same order, so are we also bound to study both on the same scientific principles, and to bend our minds to the one as to the other, in recognition of their origin in the supreme will and wisdom of nature, and in the order of the universe.

It is impossible to lay too much stress on these truths, for they are not generally admitted, though universally applicable. Superstitions of old times, the popular prejudices of an incoherent and dead, but not buried philosophy, the idols of the tribe, the den, the market, and the theatre, as Bacon enumerates them—all these are in the way to obstruct true industry, to crush simplicity, and to bolster up mysticism.

A second application of the inductive system of philosophy to the study of diseases, consists in the recognition of a unity of cause for every great natural fact. This rule follows as a necessity upon the preceding one. For there is no phenomenon in all nature which admits, when fully understood, of being referred to, or explained by, *two* causes. And if in any given case two or more causes are supplied, the one of which seems as satisfactory as the other, it is beyond all dispute that the true discovery of the cause of the phenomenon has not been yet alighted on. The affirmative may, it is true, be in one of the causes determined upon, but until the other assumed causes are excluded, or in other words turned into negatives, the discovery is unrevealed.

But in medical investigations even this simple and obvious mode of reasoning is often, if not generally, ignored; and one is constantly finding those who are presumed to be our closest observers, assigning two, three, or even four causes to one disease, so that it becomes in the end a rule, in speculating on the origin of a disease, to speak of its causes and not of its cause.

This is the very error into which the induction of Aristotle was twisted by his followers. And it is owing to this mistake that, for so many men, so many hypotheses as to cause have arisen. One man, speaking of typhus, says that its origin is atmospheric; another thinks it is due to a deficiency of ozone; a third that it is from a specific poison caused in various ways, and carried by water or air; a fourth, that it is a disease of the body *per se, i. e.* individual; while a fifth, least consistent of all, is content to accept the idea that all these causes may have their influence, that they club together to get up the exhibition.

Thus, in regard to this one disease as the type of all, the floundering business goes on; there are half a dozen affirmatives assigned, whereas there cannot be more than one; and discovery lingers because the process of eliminating the unit cause, and of throwing the other assumed ones aside as negatives, or as mere coincidences, remains unachieved.

In saying this, it would not be just to assume that no positive facts have been arrived at by the system of simple observation and enumeration of facts, without the trouble of the exclusion process. But the instances of this sort are rare, and when found they are discovered to have been long hidden, though most obvious, and at last accidentally seen. We know for example, in these days, that syphilis spreads by the transmission of a specific poison from person to person—a truth which has been arrived at by the continued observation of vast numbers of cases. We know, too, that small-pox virus, passed from person to person by inoculation, may propagate the disease; that the bite of a dog gives rise to hydrophobia, and so on; but these are accidentally discovered facts, and do not prove anything for or against any system of philosophical research.

The application of the exclusion principle in relation to diseases extends into these branches, viz.: the study of the cause of the disorder; the study of its nature; the study of the treatment. On the first only of these three, viz., that relating to cause, we shall here dwell.

To institute a rigid inductive inquiry into the cause of any disorder, the cause itself being at present unknown, implies, doubtless, great difficulties; but we believe that such an inquiry might be so conducted as to lead to certain success. We do not of course speak of first or efficient causes, for these are out of reach, but of secondary causes or those which are traceable.

In considering an inductive inquiry of the kind here named, the following points are to be recognised :—

1. That the series of phenomena, which grouped together constitute a special disease, have a cause.

2. That this cause is a unit.

3. That at the commencement of the inquiry this cause must either be within cognisance or not within cognisance.

4. That if it is within cognisance, and yet is not known beyond doubt as the cause, the absence of such knowledge is due to the fact, that the said cognisable cause, which is affirmative, has not been isolated from other supposed causes which are negatives.

5. That a true cause must have the following elements. It must be antecedent to the effect. It must be sufficient in itself to explain the phenomena. It must respond to repeated observation. It must respond to direct experiments. It must be recognisable by the senses.

If in any given case a cause can be worked out by these rules, the inductive philosophy has triumphed, and an absolute demonstration has been supplied.

In order to arrive at this ultimatum, the present vague and acknowledged unsatisfactory modes of research must be entirely laid aside, and must be replaced by these principles :—

An acknowledgment of the uniformity of nature.

A careful and extended line of observation directed to the phenomena themselves as they appear in nature; and to the natural agencies which may be presumed to lie at the root of the phenomena.

An experimental inquiry, conducted independently of the natural phenomena observed.

The first of these three principles must be accepted as a matter of faith, based on a reasonable and careful observation of nature as a whole, and on the stability and consistency of all her manifestations. This is a matter beyond dispute; it is most obvious to those who have most knowledge; it is doubted only by the superficial and the ignorant.

The second of these principles must be based on human industry. It demands the abnegation of all assumption or faith. It calls for individual knowledge acquired by the senses, and includes many forms of knowledge and inquiry.

Under this head is embraced the subject of historical investigation. Did the phenomenon occur in any particular part of the globe, in any particular race, at any particular season of the year, during any political crisis or calamity? Did it commence at once at various points, or in one individual? Whence did it become transplanted, and by what agency—by land, river, or sea transport? and so on.

Again the phenomena of the disease as they occur to the observer himself and his cotemporaries have here to be considered. The statistics must be collected. And these statistics must include everything that can be conceived as having relation directly or indirectly to the disease, and not only to the disease, but to life and health altogether. They must include particulars which relate to the persons affected with the disease, their occupation, their mode of life, their age, their sex, their habitation, their food and their drink ordinarily and immediately before the commencement of the disorder, and their previous health, the circumstances under which their first symptoms occurred, their exact symptoms during the attack, (for sometimes, during an epidemic especially, other cases of disease are ranked hurriedly with those of the general type), and the medical treatment pursued, not so much with reference to the utility of the treatment as with reference to its effects; for it has occurred that all the original symptoms of an epidemic have been lost in the symptoms induced by the use of presumed remedial measures.

From the individual himself the inquiry must also extend to the external agencies by which he is surrounded, and to the exposures to which he has been subjected. Has he been in contact directly or indirectly with the other sick? has he been exposed to peculiar atmospherical influences? are these general atmospherical influences peculiar? and, if so, have the same peculiarities been marked out in previous periods as connected with the development of the same disorder?

Lastly, the conditions and the positions of those who escape the disorder must be carefully considered, and placed side by side with the conditions and positions of those who have suffered.

These statistical facts, moreover, must be applied to every case under the observer's notice, or the desired end may be lost in the omission. For, in truth, no man makes a fair comparison of what he has seen, unless he compares *all* that be seen, quite independently of any differences which may at first sight occur to the mind.

The very existence, indeed, of an apparent contradiction, should not lead to omissions, inasmuch as in nature a seeming contradiction often proves to be the firmest evidence of truth on further and deeper inquiry.

Whenever an observer meets, in fact, with what seems a contradiction in nature, he meets with a useful and all-important lesson, viz., that he himself is either short-sighted or on the wrong beat; so that he had better, therefore, go over his argument again, wait for further light, or pursue a new course. One of Sir Isaac Newton's grandest discoveries lay quiet for a long season, and was almost given up in despair, though in itself correct, in consequence of his having tried to prove it on an incorrect computation as to the size of the earth.

To return: the statistics of a disorder having been carefully drawn out, they next lie open for analysis. From them the cause has to be found. And now the first step is not to assume any observed fact as a cause, but to strike out unscrupulously all those observations which neither in themselves supply causes nor lead to the idea of causes. If this were carefully done in every instance, the cause of different diseases might be brought down to very narrow limits, and the Baconian system would be strictly carried out; *i. e.*, in Bacon's words, "nature would have been separated by proper rejections and exclusions." Should the result be that *all* possible causes have been excluded, it would follow obviously that the observations made in this special instance had failed; and that further inquiry was needed when the opportunity for conducting it should again occur. If in the result two or more presumed causes hold place, they would have to be reduced by a continuance of the same process, or by further observation. If the one cause were found, and answered to the name of true cause, while all other assumed causes were distinct negatives, or were seen only to be subsidiary to the true cause, the discovery would have been made.

It is to be confessed that this mode of research is laborious and even distasteful, but it has the advantage of approaching to the point, while the opposite course, which is the only one pursued even in the present day despite the great Chancellor's instructions, leads direct away from the point, into generalisations innumerable, and leaves discovery to the chance of accident or adventure.

This point is one which cannot be urged too strongly at the present moment; for surely there never was a time when statistics of disease were so much run after. But of what use are statistics, if begotten with a bias; or if misapplied, after being put together in honesty?

We look upon a man who collects statistics of disease for the purpose of supporting a preconceived view as absolutely a dangerous man, even though his results shall by accident be correct in the end; for such a man is not investigating nature at all, but is trying to seduce her—a trick which rarely succeeds, for nature is too old a dame to yield up her charms by stealth, or to be carried off by a *coup d'amour*.

The third method of inductive research, viz., induction by experiment, is, perhaps, less applicable in regard to studies bearing on the origin of diseases than it is to others of a more material kind. Still, an entirely new line of inquiry of a physiological character is open to the patient and industrious.

In regard to the production of diseases by physiological means, we labour under the disadvantage of having few animals that are susceptible to the same diseases as man. We therefore have not the subjects for direct experiment.

Finally, the more perfect our science of general physiology, resting on its part on induction, becomes, the more easily is the line drawn which distinguishes between health and disease, and the more competent are we to trace out the connection that exists between assumed cases of diseases, and the symptoms by which those are characterised. Every new and great physiological truth carefully recorded must at some time or another assist the practical physician; and it may be that in some such inquiries a physiological invention, as yet unseen, may suggest itself to meet and conquer a difficulty otherwise insurmountable; since, to take another aphorism from the *Novum Organum*, "It would be madness and inconsistency to suppose that things, which have never yet been performed, can be performed without employing some means hitherto untried."

In bringing forward this subject, we have felt fully alive to the difficulty of dealing with questions of so abstract a nature, and to our own incompetency for making them sufficiently learned on the one hand, or sufficiently simple on the other.

The subject has demanded some extension of thought and some research, and we feel least fear of criticism from those who, having studied it most attentively, are best acquainted with the unavoidable anxiety and labour connected with such a task.

We have felt, however, very strongly the importance of directing the attention of medical inquirers to the points now discussed, and it surely can do no harm to sacrifice these few pages in diverging from the ordinary course of things to consider the questions which this article suggests. Are the observers in medicine laying out their hard earnings to the best account? Is their bank safe; and will that which they are now hoarding up with miserly care be accepted in after time as good money, true coin of the scientific realm; or as so much waste paper, interesting, perchance, as fractions of an antiquarian's literary museum, but lost to the world at large, and dead with the present generation?

---

